# Human cellular restriction factors that target HIV-1 replication

**DOI:** 10.1186/1741-7015-7-48

**Published:** 2009-09-16

**Authors:** Klaus Strebel, Jeremy Luban, Kuan-Teh Jeang

**Affiliations:** 1 Laboratory of Molecular Microbiology, NIAID, the National Institutes of Health, Bethesda, Maryland, USA; 2 Department of Microbiology and Medicine, University of Geneva, Geneva, Switzerland

## Abstract

Recent findings have highlighted roles played by innate cellular factors in restricting intracellular viral replication. In this review, we discuss in brief the activities of apolipoprotein B mRNA-editing enzyme 3G (APOBEC3G), bone marrow stromal cell antigen 2 (BST-2), cyclophilin A, tripartite motif protein 5 alpha (Trim5α), and cellular microRNAs as examples of host restriction factors that target HIV-1. We point to countermeasures encoded by HIV-1 for moderating the potency of these cellular restriction functions.

## A brief overview of HIV-1/acquired immune deficiency syndrome (AIDS)

AIDS was first recognized nearly 30 years ago. The initial description was followed shortly by the discovery and characterization of its causative agent, the human immunodeficiency virus, HIV-1. Today, approximately 33 million people worldwide are infected with HIV. Each year, 2.5 million people become newly infected and 2 million others die from AIDS. While there are several effective drugs for treating HIV/AIDS, ongoing attempts to develop a useful HIV-1 vaccine and a protective antiviral microbicide face significant challenges and seem unlikely to be successful in the near future [1]. In this setting, a fuller understanding of the innate restriction mechanisms in human cells that modulate HIV-1 replication is worthwhile.

HIV-1 infects CD4+ T-cells. The virus encodes nine genes; three are regarded as 'structural' genes (Gag, Pol, Env), while the other six are considered 'accessory' genes (Tat, Rev, Nef, Vpr, Vpu, Vif). Steps in HIV-1 replication, including the interaction of the viral envelope protein (gp120) with the cellular CD4 receptor, reverse transcription to generate proviral DNA, integration, RNA transcription, viral protein synthesis, virion assembly and egress have been reviewed elsewhere [2-5]. Here, we discuss in brief the recent findings on apolipoprotein B mRNA-editing enzyme 3G (APOBEC3G), bone marrow stromal cell antigen 2 (BST-2), cyclophilin A, tripartite motif protein 5 alpha (Trim5α) and cellular microRNAs (miRNAs) as examples of host restriction factors [6-8] that target intracellular HIV-1 replication.

## APOBEC and Vif

APOBEC3 (A3) genes are unique to mammals and encompass a family of cytidine deaminases that are now believed to play an important role in the intrinsic or innate host immune response to control retroviruses, retrotransposons, hepadnaviruses, foamy viruses and, perhaps, even some DNA viruses such as human papillomavirus (reviewed in [6,9]). A3 genes have arisen through gene duplication and their number varies from one gene in mice to seven genes in humans [10]. They contain either one or two zinc coordinating domains. In enzymes containing two zinc coordinating domains, generally only one (in most cases it is the C-terminal domain) is catalytically active.

All of the A3 genes are catalytically active. However, there is an ongoing discussion on the functional importance of A3 catalytic activity for antiviral effects. For instance, the inhibition of parvoviruses and retrotransposons by A3A was found to be deaminase-independent [11-13]. Deaminase-independent inhibition by A3G was also reported for other viruses such as HTLV-1 and hepatitis B virus [14-17]. Finally, A3G and A3F were shown to inhibit HIV replication in a deaminase-independent manner (reviewed in [6]). However, most of the data supporting deaminase-independent mechanisms result from a transient overexpression of A3 proteins or are based on *in vitro *assays. Indeed, there is strong evidence that HIV-1 restriction does require A3G deaminase activity when the protein is not transiently overexpressed [18-20]. A3G is a powerful inhibitor of HIV-1 and several studies showed that only a few molecules of packaged A3G are sufficient to inhibit virus replication [20,21]. On the other hand, the inhibition of HIV-1 replication appears to require a minimum A3G threshold level. This is suggested by the observation that HIV-1 carrying a partially defective Vif gene was found to replicate, albeit with delayed kinetics, in A3G expressing CEM cells, a human cell line originally isolated from an acute lymphoblastic leukemia [22]. Under those conditions, viral DNA showed clear evidence of hypermutation whereas viral RNA was largely unaffected, suggesting a mechanism of purifying selection [22].

**Figure 1:**
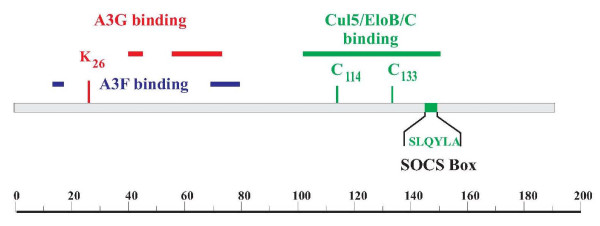
**Protein interaction domains in HIV-1 Vif**. The regions responsible for interaction with A3G (red) and A3F (blue) are discontinuous. They overlap but are not identical. The region important for interaction with the Cul5 ubiquitin ligase complex is marked in green and includes a conserved histidine-cysteine-cysteine-histidine motif and a suppressor of cytokine signaling Box domain. The scale at the bottom indicates amino acid positions.

A3 proteins are packaged into viral particles through an interaction with viral Gag protein and viral or cellular RNA [23]. Vif neutralizes the antiviral activity of A3G and A3F by inhibiting their packaging into viral particles. This involves a proteasome mediated degradation of A3 proteins as well as the degradation-independent mechanism(s) [24]. Endogenous A3G appears to be much less sensitive to degradation by Vif than transiently expressed protein [25]. While the relative contribution of degradation-dependent or independent mechanisms is still being debated, it is generally accepted that the inhibition of A3G and A3F by whatever mechanism involves a direct physical interaction with Vif (Figure 1). The regions in Vif, important for binding to A3G and A3F, appear to be overlapping but not identical. They map to the N-terminal region of Vif and appear to be discontinuous [26-31]. Interestingly, the regions in A3G and A3F important for interaction with Vif are also distinct. In A3G, residues of 126-132 in the N-terminal region were found to be sufficient for Vif binding, while Vif appears to interact with a C-terminal region of the A3F encompassing residues 283-300 [32-34]. In fact, that A3F region is sufficient to enable Vif binding and A3F degradation, whereas degradation of A3G by Vif requires a region larger than that necessary for Vif binding [32].

The structural determination of full-length A3G has so far been unsuccessful. This is mostly due to the insolubility of the purified protein. Structural data are currently available only for monomeric forms of the C-terminal catalytic domain [35-38]. However, biochemical analyses demonstrated that A3G forms oligomeric structures that are mediated or stabilized by RNA [39-41]. Huthoff *et al*. have identified oligomerization mutants of A3G that were packaging incompetently and had lost their antiviral activities [41]. Opi *et al*., on the other hand, identified an oligomerization mutant that remained packaging competent and retained antiviral activity [39]. Thus, even though A3G is likely to be recruited into viral particles as an oligomer [42], A3G oligomerization does not appear to be critical for antiviral activity, at least in transient expression assays.

One of the intriguing properties of A3G is its ability to assume different intracellular configurations. In activated cells and T cell lines, A3G exists as a mixture of low molecular mass (LMM) and high molecular mass (HMM) complexes, and it has been proposed that only the LMM form of A3G has antiviral activity [43]. A3G exerts its antiviral effect during the early phase of virus replication. In order to do so, the protein has to be packaged into virions from the virus-producing cell. A3G expressed in target cells does not generally inhibit virus replication. A notable exception is resting CD4+ T cells. In these cells A3G exists predominantly in the LMM configuration and was shown to impose a potent post-entry restriction on HIV infection [43]. This view was recently challenged by Kamata *et al*. who could not confirm the involvement of A3G in the post-entry restriction of HIV-1 [44]. Thus, the relative contribution of A3G to the resistance of quiescent T cells to HIV infection remains to be resolved.

## Vpu and BST-2

It is generally accepted that Vpu regulates the detachment of otherwise complete and infectious virions from the cell surface [45,46]. Interestingly, the ability to enhance virus release is not unique to Vpu. In fact, some HIV-2 isolates have been known to encode a Vpu-like activity in their Env glycoproteins [47-50]. Intriguingly, a similar Vpu-like activity has now also been shown for the Nef protein of several simian immunodeficiency viruses, including SIVmac and SIVagm [51,52].

Vpu-dependent virus release is host cell-dependent [53]. Indeed, several other host factors have been identified whose overexpression was associated with reduced virus release. These include the Vpu binding protein UBP [54], TASK-1, a human potassium channel protein, [55] and the recently identified host factors BST-2 (also referred to as tetherin, CD317, or HM1.24 [56,57]) and CAML (calcium modulating cyclophilin ligand) [58]. Among those, BST-2 is of particular interest since its cell type-specific expression most closely matches that of Vpu-dependent cell types. Silencing the BST-2 expression in HeLa cells by siRNA or shRNA (short hairpin ribonucleic acid) rendered virus release from these cells Vpu-independent [56,57].

A functional Vpu-BST-2 interaction was first reported in a quantitative membrane proteomics study where Vpu expressed from an adenovirus vector was found to reduce cellular expression of BST-2 in HeLa cells [59]. Intriguingly, BST-2 expression is cell type dependent and is induced by interferon treatment [56,57,60,61] which is consistent with the previous observation that cell lines that did not normally require Vpu for efficient virus release became Vpu-dependent upon being treated with interferon [60].

**Figure 2:**
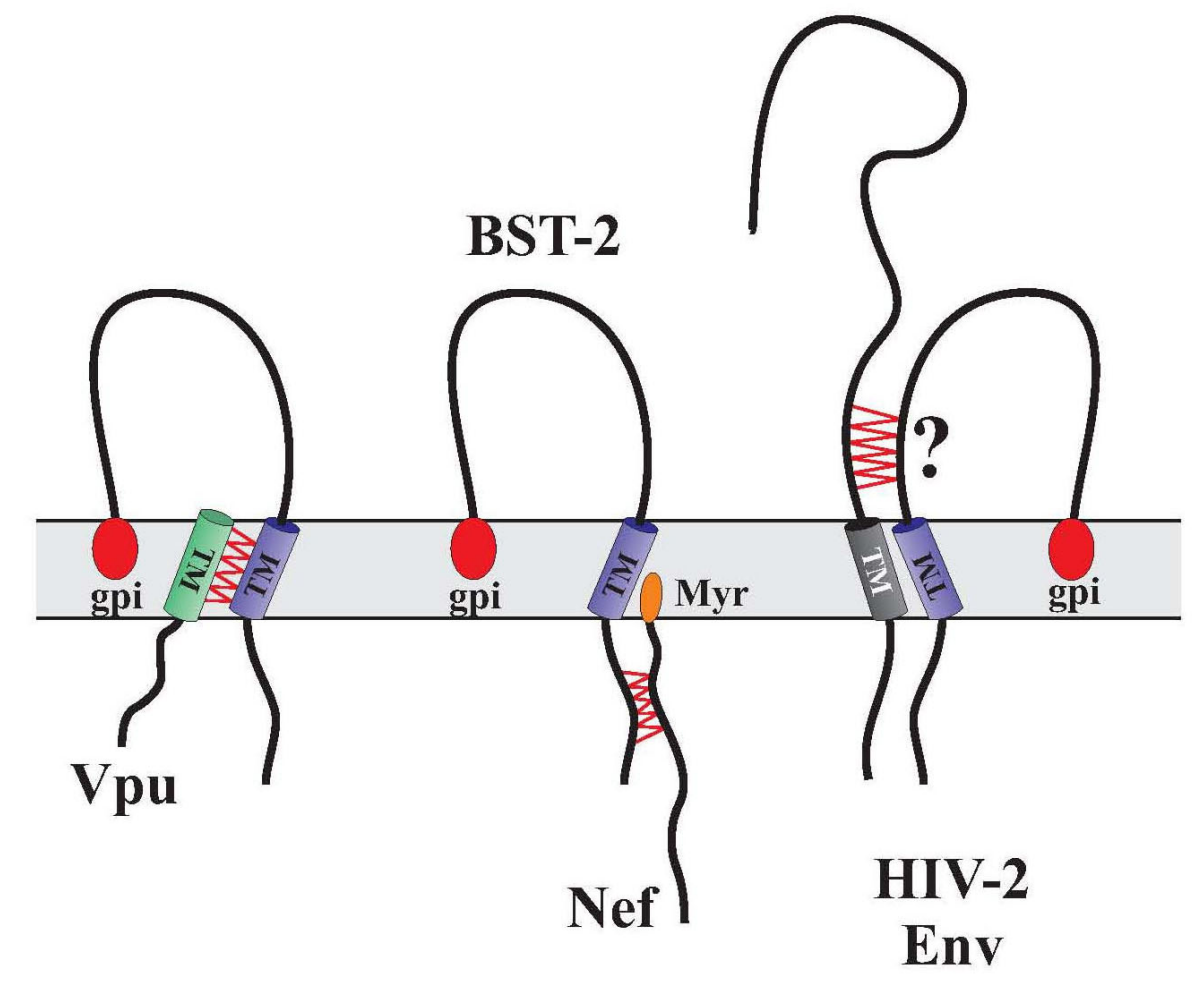
**Interaction of BST-2 with HIV-1 proteins**. Vpu interacts with BST-2 through its transmembrane domain. Interaction of BST-2 with Nef involves the cytoplasmic domain. The interaction of HIV-2 Env with BST-2 has not been mapped, but may involve the ectodomain. Proposed interaction points are indicated by red zig-zag lines. Myr: Nef myristoylationl; gpi: glycosyl-phosphatidylinositol anchor.

How does BST-2 inhibit virus release? BST-2 is a 30-36 kDa type II transmembrane glycoprotein [62]. The protein has both an N-terminal transmembrane domain and a C-terminal glycosyl-phosphatidylinositol (GPI) anchor [63] (Figure 2). This has led to a model, in which BST-2, by means of its N-terminal transmembrane domain and its C-terminal GPI anchor, tethers otherwise fully detached virions to the producer cell [56]. Such a model is consistent with the observation that Vpu-defective particles can be released by protease treatment [46] or by physical force [45]. Particles released by the latter method were found to be fully infectious [45]. The tethering model is elegant and simple but it still awaits formal experimental validation. Somewhat unexpectedly, Vpu-deficient virions that were separated from the cell surface by physical force did not contain detectable amounts of BST-2 [61] even though the electron micrograph data imply that BST-2 not only tethers particles to the virus producing cell but crosslinks virions among each other [45,56].

How does Vpu counteract the BST-2 imposed restriction of virus release? Recent data suggest that the BST-2 transmembrane (TM) domain is critical for interference by Vpu [64-66] consistent with the previous observation of the importance of the Vpu TM domain for the regulation of virus release [67-69]. This suggests a direct physical interaction of Vpu and BST-2. Indeed, Vpu was found to interact with BST-2 in HeLa cells [70]. It is unclear how Vpu inhibits BST-2. One simple way of interference would be to down regulate the protein from the cell surface. Indeed, several studies have noted that Vpu can down regulate BST-2 [57,61,71] although Vpu does not seem to increase the rate of BST-2 endocytosis [72]. This suggests that Vpu may affect the resupply or the surface delivery of BST-2, a function that could be exerted from an intracellular location such as the trans-Golgi compartment [70,73]. Interestingly, Vpu was found to reduce total cellular levels of endogenous as well as exogenously expressed BST-2 [59,61]. How this is accomplished is not yet completely clear. However, it has been suggested that antagonism of BST-2 by Vpu involves proteasomal degradation by Vpu [74,75]. It was also reported to encompass a β-TrCP (beta transducing repeat containing protein)-dependent endo-lysosomal pathway [70,72]. The involvement of β-TrCP in the virus release activity of Vpu necessitates the conservation of Vpu's TrCP binding motif. However, mutation of this motif was previously found to only partially affect the virus release activity of Vpu [57,76,77] and in more recent studies the mutation of the TrCP-binding site in Vpu (Vpu26) did not affect replication kinetics in a variety of cell types, including the peripheral blood mononuclear cells [61]. Expression of the TrCP binding-deficient Vpu26 had no effect on BST-2 stability. To the contrary, it appeared to stabilize or even increase the BST-2 surface expression [61]. Thus, more work needs to be done to sort out which of the effects of Vpu on BST-2 (surface expression, degradation or intracellular sequestration) are critical for enabling enhanced virus release.

The effects of BST-2 are not restricted to lentiviruses. They have also been shown to affect Ebola virus release [78,79]. Furthermore, the inhibition of HIV-1 virus release is not limited to human BST-2 but is also observed in mouse, rat and monkey BST-2 [65,74]. However, only the human BST-2 is sensitive to Vpu. Finally, the ability of HIV-2 Env and SIV (simian immunodeficiency virus) Nef to antagonize BST-2 appears to involve cell surface down modulation of BST-2. However, unlike Vpu, neither Nef nor HIV-2 seems to affect the stability of BST-2 [51,70]. Although Vpu, targets the BST-2 TM domain, Nef targets the cytoplasmic domain (Figure 2) [51,52]. The site(s) in BST-2 critical for its sensitivity to HIV-2 Env have not yet been mapped. Recently, however, it was noted that the Vpu-like activity of HIV-2 Env was regulated by a single amino acid difference in the ectodomain of its TM subunit [80]. It will, therefore, be interesting to see if HIV-2 Env targets the BST-2 ectodomain.

A final point concerns the question of whether or not BST-2 deserves the label of restriction factor. It is true that BST-2 potently inhibits the release of viral particles and that there does seem to be a selective pressure to maintain Vpu expression, at least in the SHIV **(**simian-human immunodeficiency virus) model [81]. However, Vpu is not absolutely essential for viral replication and there are primary HIV-1 isolates that lack a functional Vpu gene due to the mutation of the initiation codon [82]. The inhibition of Vpu will reduce virus release but that does not prevent viral spread. Instead, virus replication will simply shift from a cell-free mode to a cell-to-cell mode. This finding likely explains why Vpu-deficient viruses replicate in tissue culture with the same kinetics as wild type virus [45,83-85]. Thus, BST-2 is a restriction factor in the sense that it limits the release of viral particles from infected cells. However, it may not be a restriction factor in the sense that it blocks the spread of virus in an infected individual.

## Cyclophilin

In the early 1990s cyclophilin A (Cypa) was reported to be a cellular factor that binds HIV-1 capsid protein (CA), but not the CA of other lentiviruses such as SIVmac239 [86-88]. Recently, it was shown that it also interacts with the CA of some HIV-2 isolates, with SIVagmtan and with FIV (feline immunodeficiency virus) [89-91]. Cypa is a small globular protein that binds to proline-containing peptides via its hydrophobic pocket and is believed to have subtle, but global, effects on protein folding within cells. Among the specific cellular functions of Cypa, it regulates the nuclear export of Zpr1 (zinc finger protein 1) in yeast [92] and the kinase activity of Itch (inducible T-cell kinase) in CD4+ T cells [93]. As a complex with the immunosuppressive drug cyclosporine, it is a potent inhibitor of the CA-dependent phosphatase calcineurin in T cells [94]. Interaction with HIV-1 CA does, indeed, involve a critical proline residue [95] and binding of CypA catalyzes a conformational change in CA [96].

**Figure 3:**
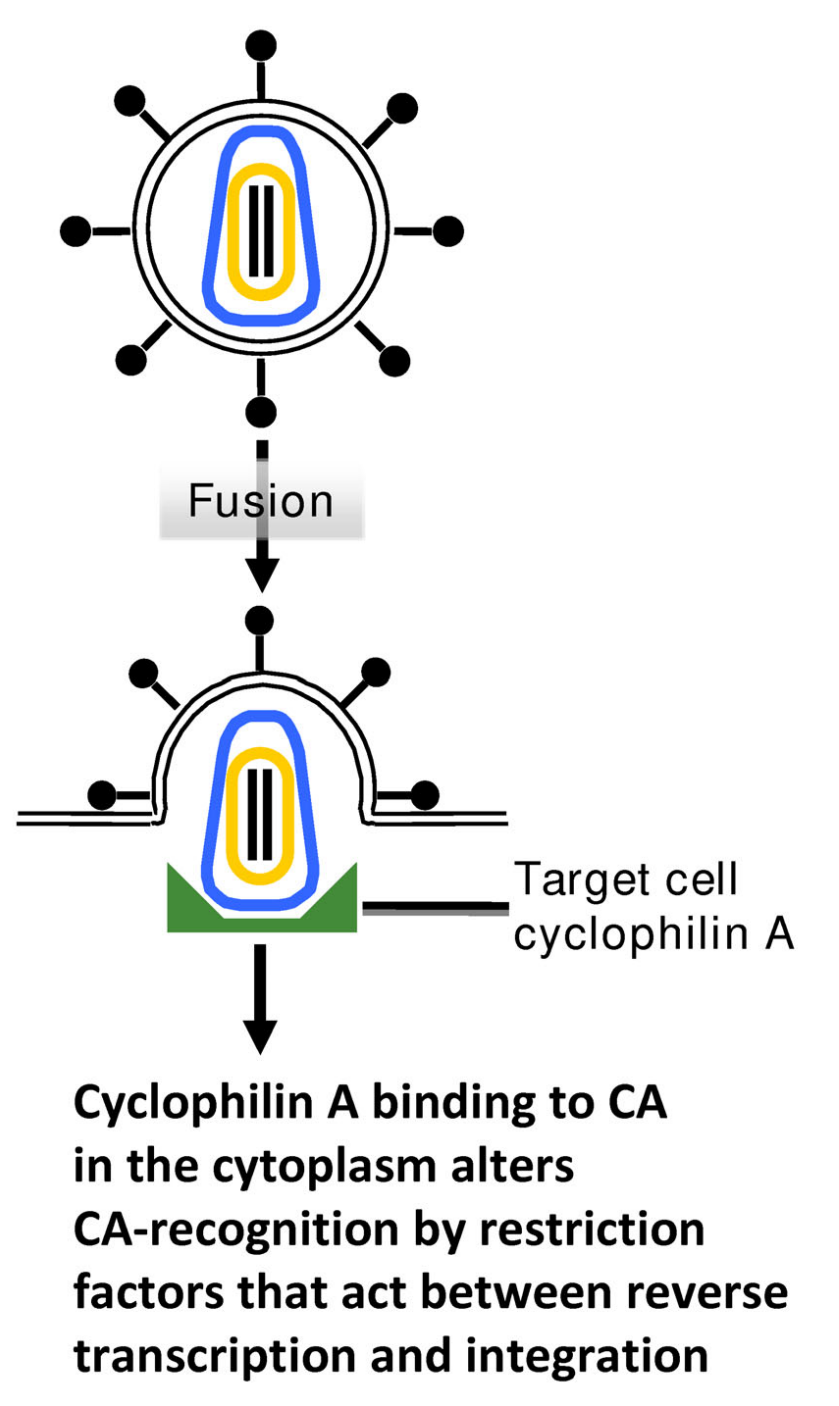
**The effect of host cell cyclophilin A on HIV-1 transduction**. Host cell cyclophilin A (shown in green) binds to the HIV-1 CA and regulates CA-recognition by host cell restriction factors. In some cases, cyclophilin A promotes HIV-1 CA recognition by these factors (for example, by rhesus macaque TRIM5alpha). In other cases, human cells for example, cyclophilin A seems to prevent recognition by these factors. The effect of cyclophilin A is, therefore, determined by the effect of the restriction factor, which may act at the time of reverse transcription, nuclear import, or integration.

CypA acts within the target cell soon after HIV-1 infection [95,97,98], in some cases to promote infection and in others to inhibit infection [97,99,100]. Sometimes the effect is at the level of reverse transcription [95]. At other times the effect of CypA is evident later, perhaps at the level of nuclear entry [100,101]. It is generally thought that CA indirectly regulates reverse transcription via its effects on an ill-defined process called uncoating (Figure 3). However, CA remains associated with nascent viral cDNA, may dock to the nuclear pore and does seem to be a critical determinant in HIV-1's ability to cross the nuclear pore or even to integrate into host chromosomal DNA [100,102-104]. The simplest explanation for the diverse effects of CypA is that it regulates CA interaction with host factors, either uncoating factors, nuclear pore components or restriction factors that act in a similar fashion to TRIM5 [105-107] (Figure 3).

As discussed below, characterization of the varied effects of CypA steered investigators to the discovery of TRIM5 as an innate, species-specific anti-HIV-1 factor in the cells of non-human primates [106,107]. In the future, the proviral and antiviral effects of CypA in different human cell types may guide the discovery of similar factors acting in human cells [107-110]. A more detailed structural analysis of the HIV-1 CA lattice [111] may reveal how CypA alters the CA conformation and, thus, the accessibility of antiviral factors such as TRIM5. A modest inhibition of viral load by cyclophilin A-binding drugs (non-immunosuppressive analogues of cyclosporine) was seen in some individuals with HIV-1 infection [112], leaving open the possibility that such drugs might play a role in the clinic in the future.

## TRIM5

Cells from South American owl monkeys potently inhibit HIV-1 transduction [113]. This HIV-1 restriction activity was examined in great detail because of its apparent dependence upon CypA [105-107]. These studies led to the cloning of an essential factor, the TRIM5-CypA fusion gene [106]. A similar anti-HIV-1 restriction activity in rhesus macaque cells was shown to be due to TRIM5alpha [114]. TRIM5alpha orthologues with anti-retroviral activity have been observed in most primate species, as well as in cows [115,116] and rabbits [117]. The gene is deleted in dogs and truncated in cats [118]. Rodents have multiple copies of the gene, though antiviral activity has not been demonstrated [119]. The owl monkey gene was created by retrotransposition of a CypA cDNA into the TRIM5 locus [106,120,121]. Amazingly, a TRIM5-CypA fusion allele was created by an independent retrotransposition event in Asian macaques [122-125].

TRIM5 is one of the more than 70 tripartite motif genes in the human genome. Tripartite refers to proteins bearing a RING finger, a B-box and a coiled-coil domain. The two latter domains promote TRIM5 multimerization and are almost always required for anti-HIV-1 restriction activity [126,127]. The TRIM5 RING finger has E3 ubiquitin ligase activity *in vitro *and *in vivo *[128-130] and, while not strictly required for restriction activity, it clearly plays an important role in TRIM5-mediated restriction. This apparent paradox has been explained by the finding that TRIM5 blocks HIV-1 infection at more than one step, reverse transcription and nuclear import [101,131,132].

The CypA domain in the TRIM5-CypA fusions is clearly a CA-binding determinant [106,120]. A single amino acid change in the CypA domain distinguishes the owl monkey and macaque proteins which alters retroviral restriction specificity [122]. It has been much harder to demonstrate CA-binding activity for the PRY-SPRY domain (a domain present in the Dictystelium discoideum dual-specificity kinase and the PYRIN protein gene family) of the TRIM5alpha isoform [133-135], apparently because its interaction is much weaker than that of the CypA-CA [127]. It is allele specific differences in the PRY-SPRY domain that determine the differences in retroviral restriction specificity for different TRIM5alpha orthologues [136-139].

**Figure 4:**
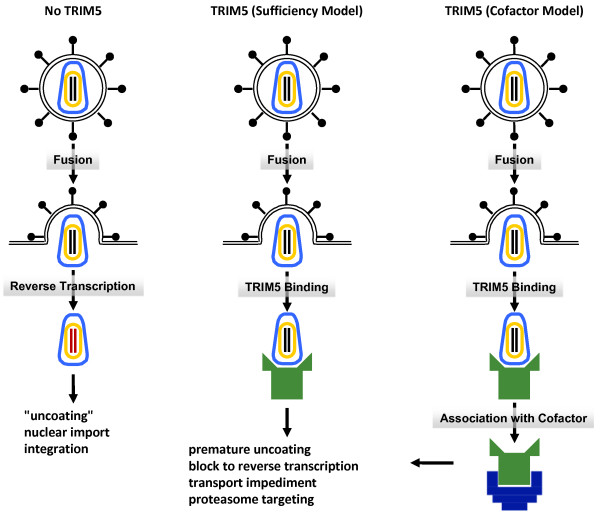
**The effect of TRIM5 on retroviral transduction**. In the absence of a restrictive TRIM5α orthologue (No TRIM5), the retroviral genomic RNA is reverse transcribed, translocated to the nucleus and integrated. In the presence of a restrictive allele of TRIM5, retroviral infection is blocked at the level of reverse transcription and, probably, also at the level of nuclear import. According to some investigators, TRIM5 binding to the incoming virion capsid is sufficient to disrupt infection (sufficiency model). According to other investigators, the antiviral effects of TRIM5 require as yet to be identified, cellular cofactors (cofactor model).

The most important question that remains unanswered about TRIM5 is how it restricts retroviral infection. Is it simply that a multimer of TRIM5 binds to incoming virion cores and prematurely uncoats them? Or is the mechanism more complicated, requiring accessory factors (see Figure 4) [107-109,140]? What, if any, is the role of ubiquitin in the restriction mechanism? What is the three-dimensional structure of TRIM5 and how does it recognize the virion core? TRIM5 forms cytoplasmic bodies but the significance of these remains unclear, especially since the endogenous protein has not been visualized.

## Non-coding microRNAs and RNA-silencing

Although restriction factors have traditionally been thought of as proteins, recent findings suggest similar roles played by non-coding RNAs. This concept seems to make sense because, while less than 2% of the human genome encode for proteins, >70% of human DNA are transcribed into non-coding RNAs (ncRNAs) [141]. NcRNAs come in several forms including siRNA, miRNA, and piRNA with each being regulated differently [142-147]. Amongst these RNAs, the best studied is perhaps the ~21-23 nucleotide long microRNA (miRNA). The biogenesis of miRNAs has been reviewed in detail elsewhere [146] and currently more than 800 discrete members are listed in the human miRNAs database (see ).

For RNA interference (RNAi) activity, miRNAs assemble into RNA-induced silencing complexes (RISC) which contain an Argonaute protein with additional RNA-binding co-factors such as TRBP (TAR RNA-binding protein) [148-150]. An active miRNA-RISC silences a target mRNA via limited, and sometimes imperfect, base-pairing between the 5'-positioned 2-8 nucleotides of the miRNA (the 'seed' region) [151] and the mRNA. Currently, it is held that a miRNA primarily targets the 3' untranslated region (UTR) of a mRNA. However, coding sequences and the 5' UTR of mRNAs can serve also as miRNA-substrates [152-154]. Indeed, a single miRNA through 'imperfect' (wobbled) base-pairing with mRNA-targets could potentially recognize and regulate the expression of up to one hundred different mRNAs [155].

## miRNA-restriction of HIV-1

**Figure 5:**
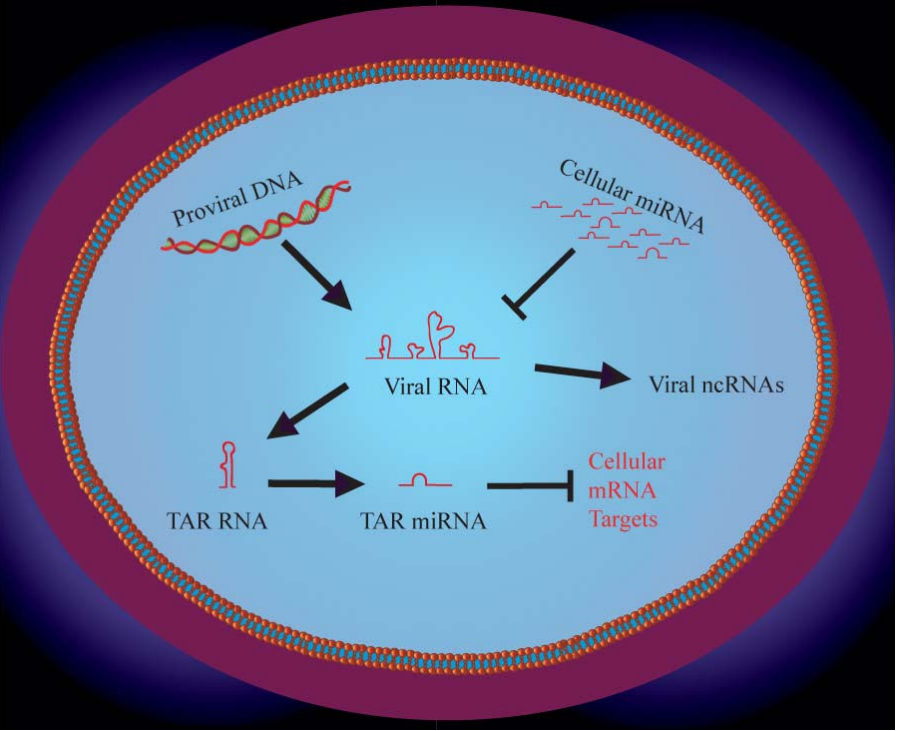
**Schematic representations of roles played by small non-coding RNAs in HIV-1 replication**. Integrated HIV-1 provirus is transcribed into viral RNA which can be targeted by cellular miRNAs. HIV-1 transcripts can also be processed into small ncRNAs that could function in cellular RNAi pathway(s). The viral trans-activation responsive TAR RNA has been reported to be processed into a viral miRNA which could act to modulate the expression of cellular mRNAs.

MiRNAs are used by plants and lower eukaryotic cells to restrict infecting viruses [156]. In higher eukaryotes, the same functional complement of miRNA components and RNA-silencing machinery are conserved. However, it has been debated whether mammals might have extinguished this ncRNA-based antiviral strategy [157-160]. To date, experimental findings, as outlined below, support the idea that mammals continue to employ ncRNAs to 'silence' viruses. First, short interfering RNAs (siRNAs) [161], piwi-interacting RNAs [142,162] and Dicer-processed miRNAs [163] have been shown to suppress mammalian endogenous retroviruses in somatic, germ and mouse embryonic stem (ES) cells. Secondly, bioinformatics analyses have extended this notion of antiviral defense to a plethora of human miRNAs that can target many types of viruses [164,165]. Specifically, miRNA-regulation of HIV-1 infection has been experimentally verified and independently reported by four groups of investigators [166-169]. Thirdly, recent results show that human cells can process HIV-1 RNAs into small ncRNAs that could potentially interdigitate into the cellular RNAi pathways [170]. Collectively, these discrete findings converge to suggest that RNAi-restriction pathways in human T-lymphocytes and macrophages are operative for HIV-1 replication (Figure 5).

## Suppression of RNAi enhances HIV-1 replication

Whether or not RNAi is a physiological mechanism for modulating viral replication can be addressed by asking what happens to an HIV-1 infection when the infected cells are attenuated for their RNAi activity. The RNAi pathway has been deliberately disabled in human cells by the knock down of either the Dicer protein [171] or the RISC components [172] and such suppressions have enhanced HIV-1 replication in cells. Separately, when heterologous RNA-silencing suppressors (RSS), such as the Vaccinia virus E3L protein, influenza A virus NS1 protein, Ebola virus VP35 protein [173] or NS3 protein of Rice Hoja Blanca virus [174], were over expressed in human cells (Table 1) HIV-1 replication in RSS-expressing cells was also considerably enhanced over that of control cells. The most simple interpretation of these results is that the RSS proteins encoded by the viruses repressed a physiologically restrictive cellular RNAi on HIV-1 and that the neutralization of this RNAi directly increased viral replication. The generality of RNAi as a mammalian antiviral mechanism is illustrated by the finding that many viruses encode RSS proteins (Table 1) and that, when cellular RNAi is suppressed, the replication of vesicular stomatitis virus (VSV) [175] and influenza A virus [176] is also enhanced. Collectively, the extant observations are consistent with a conserved mammalian ncRNA/Dicer-RISC/RNAi innate restriction pathway(s) that guards human cells against endogenous and exogenous viruses.

**Table 1:** Examples of RNA silencing suppressors encoded by several viruses.

**Virus**	**Viral Suppressors**	**References**
Adenovirus	VA RNA	***Lu 2004 ***[199]; ***Andersson 2005 ***[200]; ***Xu 2007 ***[201]

HCV	Core protein	***Chen 2008 ***[202]; ***Wang 2006 ***[203]

Ebola	VP35 protein	***Haasnoot 2007 ***[184]

Influenza A virus	NS1 protein	***Li 2004 ***[204]; ***Delgadillo 2004 ***[205]; ***Bucher 2004 ***[206]; ***Haasnoot 2007 ***[184]; ***de Vries *2009 **[186]

Nodamura Virus	NoV B2 protein	***Sullivan 2005 ***[207]

Primate Foamy Virus	Tas protein	***Lecellier 2005 ***[208]

HIV	Tat protein, TAR RNA	***Bennasser 2005 ***[182]; ***Haasnoot 2007 ***[184]; ***Bennasser 2006 ***[183]; ***Qian 2009 ***[185]

Rice Hoja Blanca Virus	NS3	***Schnettler 2009 ***[174]

[This table is modified after Grassmann and Jeang, Ref. [159]].

At what point of an infection might HIV-1 encounter cellular RNAi-restriction? Berkhout *et al. *reported that the infecting HIV-1 genome is initially sheathed [177] to repel RNAi surveillance. However, at a later stage the RNAi machinery can access and process HIV-1 RNA, as evidenced by the cellular production of virus-encoded TAR (trans-activation responsive RNA) miRNAs [178-180] (Figure 5). Nevertheless, despite the RNAi activity of the cell, in most settings, HIV-1 continues to replicate. This replication is partly explained by the action of the virus-encoded arginine-rich RNA-binding protein, Tat, which acts in an RNA-binding dependant manner [181] as an RNAi-suppressor [182] and by the viral TAR RNA which acts as an RNAi-decoy [183]. The function of HIV-1 Tat as an RNAi-suppressor has been characterized by several investigators and appears to be widely conserved in diverse experimental settings [182,184-186]. Finally, as already noted, RNAi-suppressors appear to be commonly conserved in many mammalian viruses (Table 1). Indeed, viruses such as herpes simplex do employ RNAi-suppression for their replication in human cells [187].

Cellular RNAi virus interaction has been speculated to reshape viral genome sequences overtime [157,188]. Accordingly, it serves viruses, such as HIV-1, well to evolve functions that can alter the miRNA expression profile of the cell to repress virus-noxious miRNAs and to enhance virus-propitious miRNAs. Evidence that such changes occur during viral infection has been reported [171,189,190], suggesting a dynamic strike-counterstrike interplay between the virus and the host [191,192]. Future investigations are needed in order to better understand how RNA-based antiviral restriction complements protein-based restriction.

## Concluding remarks

We have raised several examples of host cell factors that can act to impede HIV-1 replication. The emerging scenario suggests that a number of different steps in HIV-1 replication (for example, uncoating, reverse transcription, transcription and virus release) can be targeted by APOBEC3G, cyclophilin, TRIM5α, BST-2 and RNAi. Remarkably, the cellular stratagems against the virus are apparently moderated by HIV-1 encoded countermeasures (for example, Vif, Vpu and Tat). Hence, intricate strike-counterstrike interactions apparently govern the ultimate outcome of HIV-1/cell infection. The overall picture is likely to be even more complex. Recent genome-wide screenings have revealed many hundreds of host cell proteins that appear to be needed in order to activate HIV-1 replication in human cells [193-196]. Additional genome-wide screens will undoubtedly uncover an equally numerous number of host cell factors that restrict HIV-1 replication. The limited examples of HIV-1 restriction factors presented here represent a prelude to the many more restriction entities that still await identification. The next challenge is to elucidate these entities and then apply them to the development of additionally useful interventions against HIV-1/AIDS. For example, the further discovery of cellular factors which obstruct HIV-1 integration [197] could serve to aid the development of novel adjunct therapies that synergize with extant integrase inhibitors [198]. Exciting progress in antivirals lies ahead.

## Abbreviations

CA: caspid protein; CML: calcium modulating cyclophilin; CypA: cyclophilin A; Env: envelope protein; FIV: feline immunodeficiency virus; Gag: group specific antigen; GPI: glycosyl-phosphatidylinositol; HMM: high molecular mass; Itk: inducible T-cell kinase; LMM: low molecular mass; miRNA: microRNA; ncRNA: non-coding RNA; Nef: negative factor; piRNA: PIWI interacting RNA; Pol: polymerase; Rev: regulator of viral RNA expression; RISC: RNA-induced silencing complexes; RNA: ribonucleic acid; RNAi: RNA interference; shRNA: short hairpin RNA; siRNA: short interfering RNA; RSS: RNA-silencing suppressors; SHIV: simian-human immunodeficiency virus; SIV: simian immunodeficiency virus; TAR: trans-activation responsive RNA; Tat: transcriptional activator of transcription; TM: transmembrane; TRBP: TAR RNA-binding protein; UTR: untranslated region; Vif: viral infectivity factor; Vpr: viral protein R; Vpu: viral protein U; Zprl: zinc finger protein 1.

## Competing interests

The authors declare that they have no competing interests.

## Authors' contributions

KS, JL and KTJ participated equally in the writing of this review article.

## Pre-publication history

The pre-publication history for this paper can be accessed here:


